# *‘An experience of meaning’*: A 20-year prospective analysis of delusional realities in schizophrenia and affective psychoses

**DOI:** 10.3389/fpsyt.2022.940124

**Published:** 2022-08-04

**Authors:** Cherise Rosen, Martin Harrow, Clara Humpston, Liping Tong, Thomas H. Jobe, Helen Harrow

**Affiliations:** ^1^Department of Psychiatry, University of Illinois at Chicago, Chicago, IL, United States; ^2^Department of Psychology, University of York, York, United Kingdom; ^3^School of Psychology, Institute for Mental Health, University of Birmingham, Birmingham, United Kingdom; ^4^Advocate Aurora Health, Downers Grove, IL, United States; ^5^Independent, Chicago, IL, United States

**Keywords:** delusions, hallucinations, schizophrenia, affective psychosis, longitudinal research

## Abstract

Delusions are transdiagnostic and heterogeneous phenomena with varying degrees of intensity, stability, and dimensional attributes where the boundaries between everyday beliefs and delusional beliefs can be experienced as clearly demarcated, fuzzy, or indistinguishable. This highlights the difficulty in defining delusional realities. All individuals in the current study were evaluated at index and at least one of six subsequential follow-ups over 20 years in the Chicago Longitudinal Study. We assessed 16 distinct delusions categorized as thought or thematic delusions. We also examined the probability of recurrence and the relationships between delusions and hallucinations, depression, anxiety, and negative symptoms. The sample consisted of 262 individuals with schizophrenia vs. affective psychosis. Thought delusions were significantly different between groups at all follow-up evaluations except the 20-year timepoint. Thematic delusions were more common than thought delusions and show a significant decreasing pattern. In general, delusional content varied over time. Referential, persecutory, and thought dissemination delusions show the highest probability of recurrence. Hallucinations were the strongest indicator for thought, thematic, and overall delusions. The formation and maintenance of delusions were conceptualized as a multimodal construct consisting of sensory, perceptual, emotional, social, and somatic embodiment of an “*experience of meanings*”. Given the significant associations between delusions and hallucinations, future work incorporating participatory research is needed to better define and align subjective and objective perspectives. Our research also points to the need for future clinical interventions that specifically evaluate and target the coexistence and entanglement of delusions and hallucinations.

“It was as if I suddenly gained a new form of consciousness, that I discovered a different sort of world which others couldn't understand (1).”

## Background

Delusions are transdiagnostic phenomena that form part of the essential features of major psychotic disorders including schizophrenia, schizoaffective disorder, affective psychosis, and delusional disorder, and may also occur in neurological conditions such as dementia. Although it is still debatable whether delusions or delusion-like ideas are normally distributed along a continuum with everyday feelings of doubt and suspicion or are better defined as categorically different experiences, it is evident that some individuals in the general population may also present with quasi-delusional thinking (e.g., firmly held beliefs in conspiracy theories) (2–4). Delusions are highly complex and heterogeneous and can vary in intensity, stability, and dimensional attributes where the boundaries between ordinary everyday beliefs and delusional beliefs can be experienced as clearly demarcated, fuzzy, or indistinguishable, highlighting the difficulty in defining delusional realities. Many delusions can be episodic in nature, both within an episode and between episodes of an illness, and often vary in prevalence, severity, and dimensionality over an individual's lifespan. Other delusions may persist even after other symptoms (e.g., hallucinations) remit, albeit sometimes in less intense forms which may or may not interfere with daily functioning. Psychotic illnesses usually follow a remitting and relapsing course, and it can be difficult to tease out whether the delusions themselves change within an episode of psychosis, or if they will remit and relapse as episodes of illness do. The Chicago Longitudinal Study and many other research groups have clarified a broad range of relevant features that characterize these temporal dynamics (5–14). Less clear, however, is how individuals switch delusional type (referential, paranoid, grandiose, etc.) in different delusional episodes and which delusional categories are most likely to recur in the same individual, given a particular diagnosis, over a lifespan.

Early phenomenological psychopathologist Karl Jaspers was amongst the first to describe and classify delusions. He conceptualized delusions as consisting of three primary criteria: (1) a belief in which the “content is false and/or impossible, (2) it is held with unshakable conviction, and (3) is incontrovertible by rational argument or counterevidence (15–17)”. Importantly, Jaspers also highlighted limitations of this definition/conceptualization in such that it does not include nor articulate the subjective perspective of delusional beliefs: “to say simply that a delusion is a mistaken idea which is firmly held, and which cannot be corrected gives only a superficial and incorrect answer (15, 18)”. Jaspers defined primary delusions or “delusions proper” as encompassing the *delusional experience* (Wahnwahrnehmung) in which “there arises in the patient certain primary sensations, vital feelings, mood, awareness…This general delusional atmosphere with all its vagueness of content must be unbearable. Patients obviously suffer terribly under it, and to reach some definite idea, at last, is like being relieved of some enormous burden (19, 20)”. Secondary delusions or “delusion-like phenomena” on the other hand are defined as the judgment based on the *delusional experience*: “If we want to get behind these mere external characteristics of delusions into the psychological nature of delusions, we must distinguish the original experience from the judgment based on it, e.g., the delusional content as presented data from the fixed judgment which is then merely reproduced, disputed, dissimulated as occasion demands (18, 21–23).”

Kurt Schneider, building on this earlier work, developed the concept and categorization of first-rank symptoms (FRS) that identified a set of core symptoms, including elements of delusional perception and delusions of control/somatic passivity (experiences of bodily sensations imposed by an external agency), thought insertion, withdrawal, and broadcasting. Although FRS are more common in individuals with schizophrenia, they are no longer considered pathognomonic (12, 24, 25). Indeed, as influential as Jaspers' and Schneider's contributions are to the definition and categorization of delusions, there is unlikely to be a single criterion that lies at the core of the delusional experience and too much focus on narrow aspects of delusional thinking may even be counter-productive to the treatment and care of individuals with distressing delusions (26–28).

More recent phenomenological descriptions of delusional reality emphasize that in some cases, regardless of the type of delusion (e.g., referential, grandiose, thought insertion, “made” feelings), the quality of such lived experience is as if it were the same as consensual or everyday reality, “no matter how distant and ‘untrue' the patient's experience of reality appears to the clinician, it is still no less real for the patient...and what it means to be attached to one's preferred reality (29)”. However, so far there is no consensus on this issue amongst phenomenological researchers. Phenomenological psychopathologists have also framed delusions within the context of a “failure to co-constitute reality in terms of a loss of background certainties, commonsense, we-intentionality or basic trust and the subjectivistic withdrawal from shared reality that is ‘decoupled from the shared world' (30–34)”. First-person account of the experience has been described as follows: “Everything loses its familiarity. Predictability was completely gone. The king could have entered my room, so to speak, and I would have found that normal. I wouldn't have been surprised at all (1).”

There is much theoretical debate regarding the formation and maintenance of delusions. According to Maher, delusional beliefs, like normal beliefs, are developed to explain anomalous experiences, and in a delusional reality, anomalous experiences are considered the primary contributor, not defective reasoning, to the etiology of delusions (35–38). This one-factor theory posits that anomalous experiences alone are sufficient for delusion formation whereas two-factor theory posits that both anomalous experience and faulty cognition are required for delusions to be formed and sustained (39–42). Non-doxastic conceptualizations view delusions and psychopathology as not intrinsically interrelated, that delusions are not simply beliefs, and that delusional reality serves a more adaptive or protective role in the dynamic underpinnings as meaningfulness is given to the experience (43–45). It has been argued that the “individualistic source of evidence (e.g., perception, reasoning, memory)” and understanding of “deviations that give rise to delusions” are incomplete without the contextual “social source of evidence (e.g., testimonial isolation and testimonial discount)” and that delusions are “overwhelmingly socially and relationally themed” which is central and relevant to both the formation and maintenance of delusions within the social structure (46, 47). Overall, delusions are best understood as part of a complex multimodal sensory, perceptual, cognitive, emotional, social, and somatic embodiment that extends beyond the framework of a belief and into an “*experience of meanings*” that fundamentally redefines *the experience of reality* (1, 15, 48–50).

Other theorists and researchers argue that aberrancy of both perceptions and beliefs often found in delusions results from an evolving process of bidirectional influence associated with deficient hierarchical predictive error minimization (51, 52). Among the many computational models for delusion formation in schizophrenia, Leptourgos et al. provide strong support for the “circular inference model” which produces a reverberation effect between bottom-up sensory likelihood coded stimuli and top-down prior beliefs accounting for a characteristic set of both positive and negative symptoms, but which also has the paradoxical effect of protecting individuals with schizophrenia from sensory illusions (53). A review of the extensive theories and research pertaining to predictive coding extends beyond the parameters of this paper (54–60).

Many of the abovementioned studies and theories arising from them are cross-sectional in design; quantitative studies that follow the longitudinal trajectories, variability and course of delusions are less common. As mentioned earlier, many delusions are episodic whereas others may persist throughout an individual's lifetime, however, it remains unclear whether such delusions wax and wane with other psychopathologies, stay relatively stable over time, how they might interact with other symptoms and whether their trajectories are dependent on specific diagnostic categories. These questions are of both theoretical and practical relevance not only due to the renewed interests in phenomenology research which ought to view the individual and their symptoms as dynamic and heterogeneous, but also because conditions such as psychoses are frequently viewed as long-term illnesses and their impact on an individual's personal, social and vocational functioning is often longstanding and complex. One area of particular interest is the relationships between delusions and other clinical variables such as hallucinations: phenomenological accounts have argued that some delusions can be more perceptual than mere beliefs, whereas some hallucinations are more thought-like [e.g., (61)], but there has been little quantitative research to support and complement these accounts, or how delusions, hallucinations, and other symptoms might evolve over time and interact with one another.

To summarize, our previous Chicago Longitudinal Study on the trajectory of delusions have demonstrated that individuals with schizophrenia are more likely to experience increased prevalence and severity of delusional thinking after the acute phase which is associated with functional disability and that persecutory delusions have a better prognosis compared to other types of delusions (6, 7, 9, 14, 62–64). We have also reported a positive association between delusions, hallucinations, self-monitoring, and increased rehospitalization (6, 9). In this current study, we build on our previous research and examine the prevalence and trajectory of 16 distinct types of delusions categorized as either thought or thematic delusions by diagnostic category at six follow-up evaluations over 20 years (14, 49).

Given the complex ontological and etiological nature of delusional reality, this study sought to answer the following questions:

- If experienced once, what is the probability of experiencing the same type of delusion at a subsequent evaluation by diagnostic category over 20 years?
- What are the prevalence and course of thought delusions compared to thematic delusions by diagnostic category over 20 years?
- What are the relationships between delusions and hallucinations, depression, anxiety, and negative symptoms in psychosis?

## Methods

### Participants and measures

All individuals were evaluated at the acute phase of hospitalization as part of the Chicago Longitudinal Study, a prospective research program designed to study the naturalistic course of psychopathology, neurocognition, and recovery in schizophrenia and primary mood disorders (8, 12, 65–69). Individuals were then reassessed at 6 subsequent follow-ups over a 20-year period. Follow-up evaluations occurred at ~2, 4.5, 7.5, 10, 15, and 20 years. The study was approved by the IRB at the University of Illinois at Chicago (IRB# 1997-0053), and all individuals signed informed consent prior to initiation of study procedures at index hospitalization and at each subsequent follow-up.

### Measures used to assess psychopathology

The criteria for delusions for this study were derived from DSM-III: “a false personal belief based on incorrect inference about external reality and firmly sustained despite what almost everyone else believes and despite what constitutes incontrovertible and obvious evidence to the contrary. The belief is not one ordinarily accepted by other members of the person's culture or sub-culture (70).” Clinical symptoms of hallucinations, delusions, depression, and anxiety were assessed using The Schedule for Affective Disorders and Schizophrenia [SADS interview; (71)]. Negative symptoms were rated from behaviors exhibited during assessment interviews based on items from Strauss and Carpenter's Psychiatric Assessment Interview that included (1) poverty of speech, (2) flat affect, and (3) psychomotor retardation (72, 73).

We evaluated 16 distinct types of delusions at six follow-ups over 20 years that included: thought insertion, thought withdrawal, thought broadcasting, thought dissemination (mindreading), delusions of reference, “made” feelings or emotions, “made” impulses, “made” volitional acts, somatic, persecutory, self-deprecation (guilt/sin), nihilistic, grandiose, religious, sexual, or fantastic. Based on our previous research of delusion group categorization, these were further categorized as either passivity symptoms of thought delusions which overlap with Schneiderian first-rank symptoms (thought insertion, withdrawal, broadcasting, dissemination, referential, “made” feelings, “made” impulses, and “made” volitional acts), or thematic delusions (somatic, persecutory; self-depreciation; nihilistic; grandeur; religious, fantastic or sexual) (12, 49, 50). See Supplemental Materials 1 for details of methods and measures used.

### Statistical analyses

All available data for individuals with one or more follow-up and a diagnosis of schizophrenia or an affective psychosis were included in the analyses, which were carried out using SPSS 21 and SAS 9.4. Pearson's Chi-square and two-sample *t*-tests were used to compare demographic variables in individuals diagnosed with schizophrenia compared to individuals diagnosed with affective psychosis at index hospitalization. All analyses of symptoms that included SADS scores were converted to binary variables, 0 = no delusions and 1 = with delusions scored at either 2 or 3. To examine the probability of recurrence, we defined the persistence or “stickiness” of a delusion as the probability of experiencing the same type of delusion at a subsequent follow-up. As not all types of delusions were available at baseline, to maximize the usage of data, we also included all available baseline data for the following seven specific delusions: thought insertion, thought withdrawal, thought broadcast, somatic, nihilistic, grandeur and religious. To estimate this probability, for each of the delusions, we include only individuals with at least two follow-ups (including baseline) and at least one observation of this delusion. The proportion, number of follow-ups with this delusion minus 1/number of total follow-ups minus 1, were calculated as a measurement of stickiness for a specific delusion. Generalized Estimation Equations (GEE) were used to analyze the repeated measures of thought delusions, thematic delusions, and overall delusions by diagnostic group at each follow-up in individuals with schizophrenia compared to individuals with affective psychosis (74). We chose the autoregressive within-subject covariance structure (“working covariance”) AR(1) for the GEE analyses. Logistic models with GEE were used to explore the associations between delusions, hallucinations, depression, anxiety, and negative symptoms.

## Results

### Demographic characteristics

The sample consisted of 262 individuals with one or more follow-up evaluations who met diagnostic criteria for schizophrenia (*n* = *151*) or an affective psychosis (*n* = *111; psychotic depression n* = *52 and psychotic bipolar disorder n* = *59*). Table 1 lists demographic characteristics of the study sample at index hospitalization. See Supplemental Materials 2 for a detailed description of demographic characteristics.

**Table 1:** Demographic and descriptive characteristics.

	**Schizophrenia**		**Affective** **psychosis**		***p*-values**
	***n*** **=** **151**		***n*** **=** **111**		
Sex (male/female)	94/57		49/62		$X_{\left(1\right)}^{2}$ = 8.46, *p* = 0.004
Race (white/black)	105/46		81/29		$X_{\left(1\right)}^{2}$ = 0.52, *p* = 0.47
Age at first episode (≤ 23/≥24)	129/15		94/16		$X_{\left(1\right)}^{2}$ = 0.99, *p* = 0.32
	***M***	**SD**	***M***	**SD**	
Age at index hospitalization	23.56	4.75	22.96	5.17	*t*_(260)_ = 0.96, *p* = 0.34
Educational level at index hospitalization	12.67	1.94	13.34	2.01	*t*_(251)_ = 2.69, *p* = 0.008
SES (Hollingshead index)	3.12	1.43	2.95	1.38	*t*_(229)_ = 0.92, *p* = 0.36
Vaillant-Stevens	1.71	0.46	1.34	0.48	*t*_(212)_ = 5.67, *p* ≤ 0.001
Number of prior hospitalizations	1.48	1.80	1.67	2.38	*t*_(260)_ = 0.71, *p* = 0.48
Number of follow-up evaluations	3.49	1.92	3.86	1.83	*t*_(260)_ = 1.55, *p* = 0.12

*M, Mean; S.D., Standard deviation*.

### Probability of experiencing the same type of delusion in a subsequent evaluation

**Figure 1:**
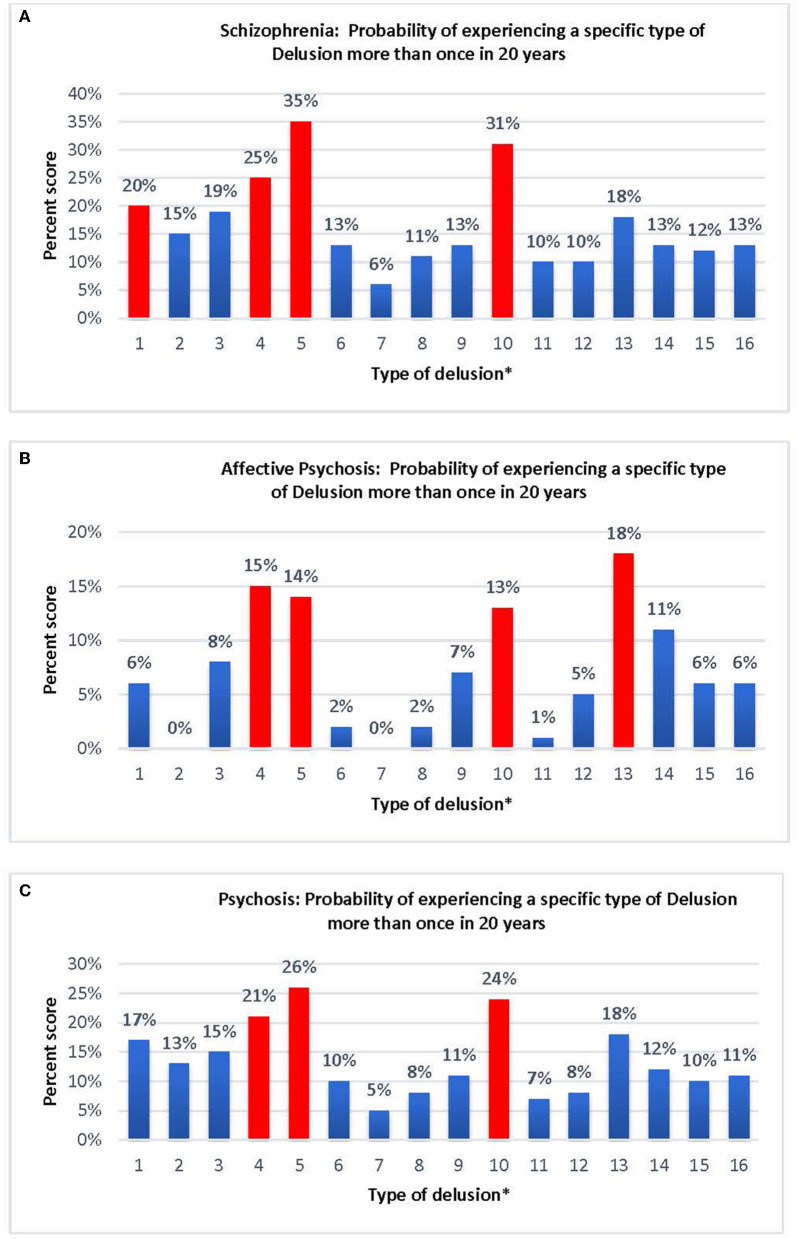
**(A)** Schizophrenia: probability of experiencing a specific type of Delusion more than once in 20 years. **(B)** Affective Psychosis: likelihood of experiencing a specific type of Delusion more than once in 20 years. **(C)** Psychosis: probability of experiencing a specific type of Delusion more than once in 20 years. [^*^Type of delusion: (1) Thought insertion; (2) Thought withdrawal; (3) Thought broadcast; (4) Thought dissemination; (5) Referential; (6) “Made” feelings; (7) “Made” impulse; (8) “Made” volition; (9) Somatic; (10) Persecutory; (11) Self-deprecation; (12) Nihilistic; (13) Grandeur; (14) Religious; (15) Fantastic; (16) Sexual].

Results of the “stickiness” of a delusion were defined as the probability of experiencing the same type of delusion at a subsequent follow-up are reported in Figure 1. Individuals diagnosed with schizophrenia show the highest probability of recurrence in referential (35%), persecutory (31%), and thought dissemination (mind-reading; 25%), followed by thought insertion (20%), whereas “made” impulses (6%) show the lowest probability of recurrence (Figure 1A). Individuals with an affective psychosis show the highest probability of recurrence in delusions of grandeur (18%), thought dissemination (15%), referential (14%), and persecutory (13%) whereas “made” impulses (0%) thought withdrawal (0%), and self-depreciation (1%), show the lowest probability of recurrence (Figure 1B). In individuals with psychosis (combined schizophrenia and affective psychoses), referential (26%), persecutory (24%), and thought dissemination (21%) show the highest probability of recurrence, whereas “made” impulses (5%) show the lowest probability of recurrence (Figure 1C).

**Figure 2:**
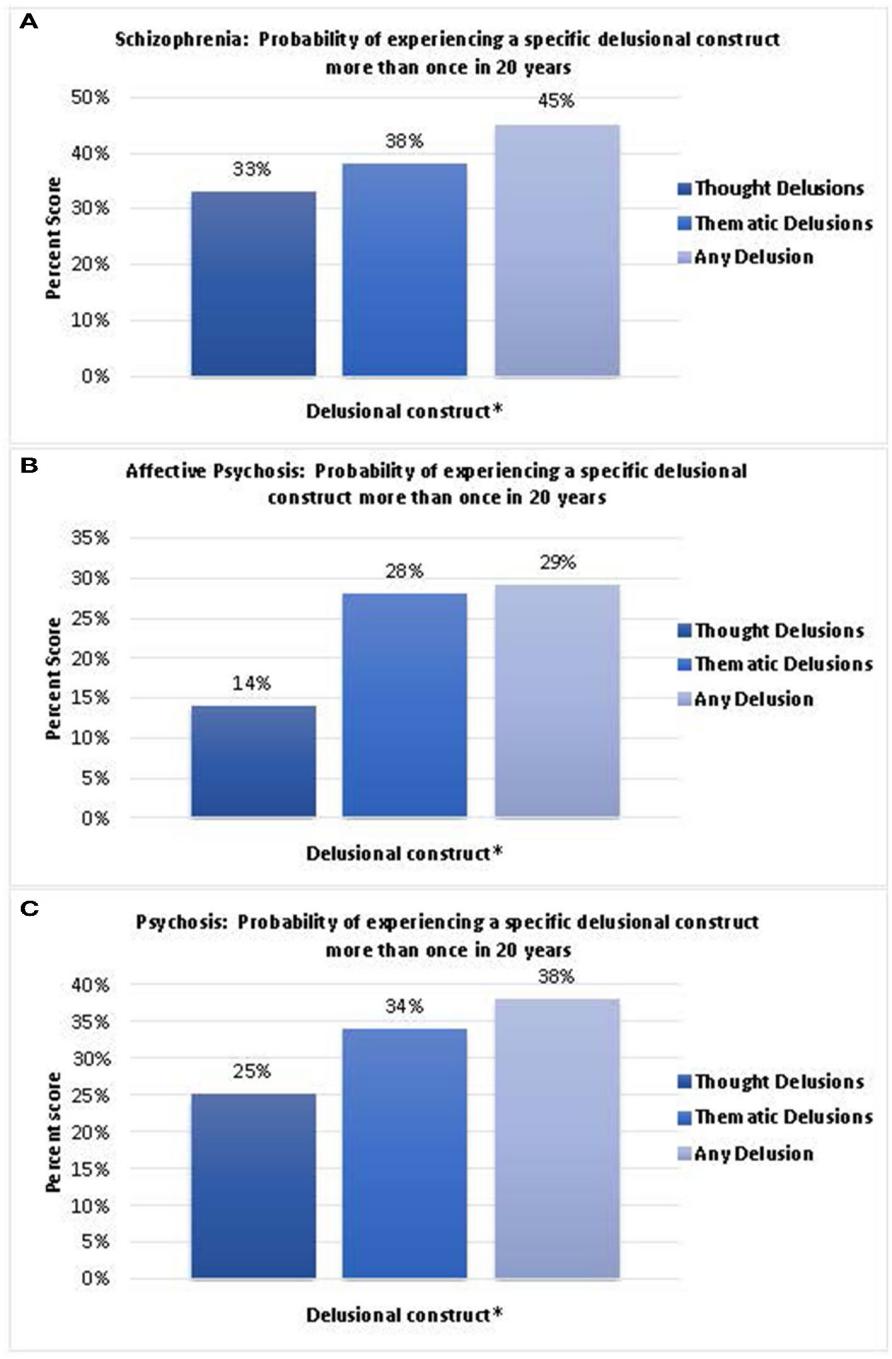
**(A)** Probability of experiencing a specific delusional construct in individuals with schizophrenia more than once in 20 years. **(B)** Probability of experiencing a specific delusional construct in individuals with affective psychosis more than once in 20 years. **(C)** Probability of experiencing a specific delusional construct in individuals with psychosis more than once in 20 years. (**Delusional construct: Thought delusion: Thought insertion; Thought withdrawal; Thought broadcast; Thought dissemination; Referential; “Made” feelings; “Made” impulse; “Made” volition. Thematic delusion: Somatic; Persecutory; Self-deprecation; Nihilistic; Grandeur; Religious; Fantastic; Sexual).

Similarly, we calculated the stickiness of the composite scores of thought (thought insertion, withdrawal, broadcasting, dissemination, referential, “made” feelings, “made” impulses, and “made” volitional acts), thematic (somatic, persecutory; self-depreciation; nihilistic; grandeur; religious, fantastic, or sexual), and any delusion (combined thought and thematic delusions) (Figure 2). Our data demonstrate that in individuals with schizophrenia, the probability of recurrence of a thought delusion was 33%, thematic delusion was 38%, and any delusion was 45% (Figure 2A). In individuals with affective psychoses, the probability of recurrence of a thought delusion was 14%, thematic delusion was 28%, and any delusion was 29% (Figure 2B). In individuals with psychosis, the probability of recurrence of a thought delusion was 25%, thematic delusion was 34%, and any delusion was 38% (Figure 2C).

### Course and trajectory of thought, thematic, and overall delusions by diagnostic category

**Figure 3:**
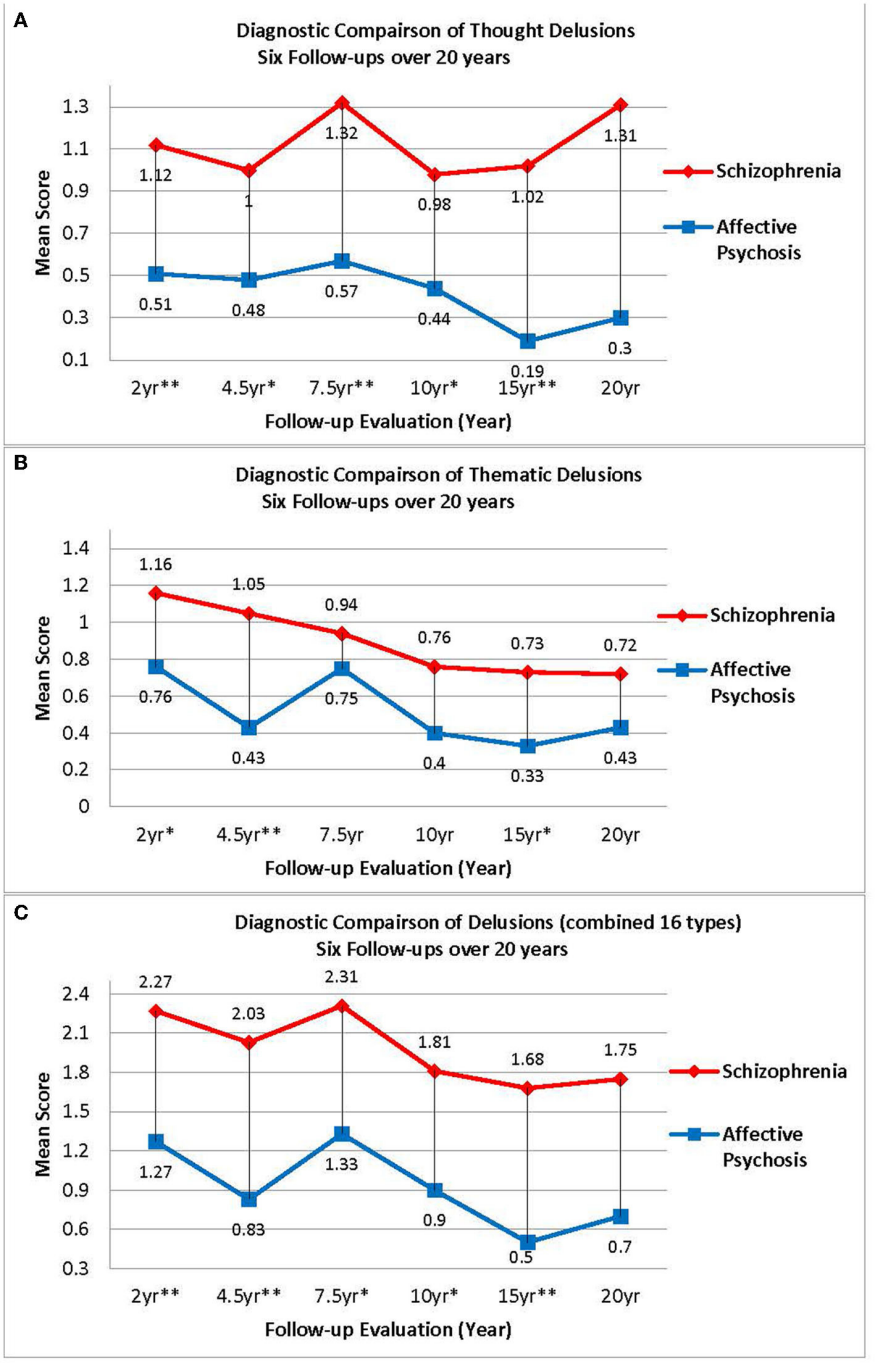
**(A)** Diagnostic comparison of thought delusions six follow-ups over 20 years. **(B)** Diagnostic comparison of thematic delusions six follow-ups over 20 years. **(C)** Diagnostic comparison of delusions (combined 16 types) six follow-ups over 20 years. [*p*-values for group effect at each time point were recorded as very significant (*p*-value ≤ 0.01, 2 stars), significant (≤ 0.05, 1 star) or insignificant (*p*-value > 0.05, no star)].

As shown in Figure 3A, the course of thought delusions stratified by each diagnostic group across 6 follow-ups show a significant effect of group [$\chi_{\left(1\right)}^{2}$ = 15.38, *p* ≤ 0.001]. Least square difference pair-wise comparisons show that individuals with schizophrenia, when collapsed across all follow-up timepoints, experienced more thought delusions compared to individuals with affective psychoses (*p* ≤ 0.001). However, the model did not show a main effect of follow-up timepoint [$\chi_{\left(5\right)}^{2}$ = 4.42, *p* ≤ 0.50]. We also examined the effect of diagnostic category and follow-up which show a significant interaction based on the linearly independent pairwise comparisons over 20 years [$\chi_{\left(11\right)}^{2}$ = 35.06, *p* ≤ 0.001]. Individuals with schizophrenia have more thought delusions at every follow-up evaluation, except for the 20-year timepoint, compared to individuals with affective psychoses (*p*'s = 0.001–0.05).

Next, we examined the course of thematic delusions stratified by diagnostic group across 6 follow-ups and found a significant effect of group [$\chi_{\left(1\right)}^{2}$ = 9.26, *p* = 0.002]. Least square difference pair-wise comparisons show that individuals with schizophrenia, when collapsed across all follow-up timepoints, experienced more thematic delusions compared to individuals with affective psychoses (*p* = 0.002). The model demonstrates a main effect of follow-up timepoint [$\chi_{\left(5\right)}^{2}$ = 17.76, *p* = 0.003]. We also examined the effect of diagnostic category and follow-up which demonstrate a significant interaction based on the linearly independent pairwise comparisons over 20 years [$\chi_{\left(11\right)}^{2}$ = 42.13, *p* ≤ 0.001]. As shown in Figure 3B, individuals with schizophrenia show more thematic delusion at the 2, 4.5, and 15-year follow-up evaluations compared to individuals with affective psychoses (*p'*s = 0.001–0.05).

Lastly, as shown in Figure 3C, we examined the prevalence of all 16 types of delusions combined by diagnostic category. The GEE model demonstrated a significant effect of group [$\chi_{\left(1\right)}^{2}$ = 13.45, *p* ≤ 0.001]. Least square difference pair-wise comparisons show that individuals with schizophrenia, when collapsed across all follow-up timepoints, experienced more delusions in general when compared to individuals with affective psychoses (*p* ≤ 0.001). The main effect of follow-up timepoint was not significant [$\chi_{\left(5\right)}^{2}$ = 10.74, *p* = 0.06]. We also examined the effect of diagnostic category and follow-up which demonstrated a significant interaction based on the linearly independent pairwise comparisons over 20 years [$\chi_{\left(11\right)}^{2}$ = 37.84, *p* ≤ 0.001]. As shown in Figure 3C, individuals with schizophrenia show more delusional activity at the 2, 4.5, 7.5, 10, and 15-year follow-up evaluations compared to individuals with affective psychoses (*p'*s = 0.001–0.05).

### Relationships between delusions, hallucinations, depression, anxiety, and negative symptoms

To explore the relationships between delusions, hallucinations, depression, anxiety, and negative symptoms, we applied fitted logistic GEE models for thematic, thought, and all delusions (See Table 2). The outcome measures for these delusions were categorized as binary variables: either present or not present at follow-up. From the fitted models, when controlling for sex and prognostic indices as measured by the Valliant-Stevens, we found that the diagnostic group and depression were not key factors in explaining the occurrence of thought, thematic, or overall delusions (combined thought and thematic), while other factors, such as the presence of hallucinations, negative symptoms, and severe anxiety were significantly associated with delusions. The presence of any hallucination was the strongest predictor for thought, thematic, and overall delusions over 20 years. The presence of negative symptoms was significantly associated with overall delusions. We also found a positive trend between negative symptoms and thought delusions, however, this finding was not statistically significant. Our data also demonstrate that severe anxiety was a strong predictor of thought, thematic, and overall delusions. Additionally, females tended to have fewer thematic delusions than males, whereas poor prognostic indicators at baseline predicted more thought and overall delusions at subsequent follow-ups.

**Table 2:** Fitted logistic GEE models for Thought, Thematic, and All delusions.

		**Thought delusions** **(AUC** = **0.9039)**		**Thematic delusions** **(AUC** = **0.8365)**		**All delusions** **(AUC** = **0.8644)**	
**Parameter**	**Level**	**Estimate**	***p*-value**	**Estimate**	***p*-value**	**Estimate**	***p*-value**
Intercept		−3.76	0.001	−2.28	0.01	−2.25	0.01
Diagnosis	Schizophrenia	0.10	0.86	0.16	0.72	0.19	0.67
	Affective psychosis	0		0		0	
Sex	Female	−0.54	0.24	−0.78	**0.05**	−0.88	**0.03**
	Male	0		0		0	
Vaillant-Stevens		1.24	**0.02**	0.72	0.07	0.90	**0.03**
**Any hallucination**		2.93	**<0.001**	2.31	**<0.001**	2.84	**<0.001**
Negative symptom		0.71	0.09	0.53	0.13	0.72	**0.05**
Depression	Mild	0.15	0.83	0.24	0.58	0.32	0.48
	Severe	−0.35	0.59	0.06	0.91	−0.10	0.86
	No	0		0		0	
Anxiety	Mild	−0.62	0.26	−0.03	0.92	−0.34	0.38
	Severe	1.53	**0.01**	1.23	**0.009**	1.30	**0.009**
	No	0		0		0	

*Bold text indicates statistical significance*.

## Discussion

Delusional realities are held in the suspension of disbelief and are given meaning associated with underlying unconscious material as a reflection of the fundamental structure, organization, and articulation of the unconscious world turned inside out and experienced as an external, unconstrained, and elaborate *experience of meaning*. Sensory and perceptual inputs are experienced by the individual from a third-person perspective where self is situated as an interlocutor navigating new, unfamiliar, and puzzling internal and external terrains. “Delusions may be irrational and even harmful, and at the same time, at the phenomenological level, they are full of meaning and provide certainty to the individual (75).” In the Spanish psychopathological tradition, there is a concept by Bartolomé de Llopis, who distinguished “living delusions” that appear in the acute phase of psychosis, from “inert delusions” that are stereotyped and lacking in conviction, and are expressed less spontaneously in a chronic or more persistent state (24, 75–78). In our study, in may be that in some instances delusions transitioned from living delusions to inert delusions as symptoms enter a more chronic state. Although we were unable to investigate their phenomenology, it is possible that these features contributed to some of the persistence and the relapsing-remitting course seen in the results. In this study we report findings from the Chicago Longitudinal Study, specifically on the prevalence and course of 16 types of delusions by diagnostic group over 20 years, and on the clinical correlates of these delusions. Understanding the types of delusions that are most experienced in individuals with schizophrenia compared to individuals with affective psychoses, and the pattern of these delusions over time, are important for efforts to design and deliver interventions targeting delusional thinking and is relevant for future work that aims to clarify mechanisms of psychosis that may be shared across diagnostic categories.

### Course, prevalence, and trajectory of thought vs. thematic delusions by diagnostic category

Although many studies have examined the prevalence and severity of delusions by diagnostic categories, this is the first study, to our knowledge, that compares the course of 16 distinct types of delusions by diagnostic category over 20 years. Previous studies have shown that specific types of delusions such as delusions of self-deprecation are more prevalent in individuals with psychotic depressive conditions, and delusions of grandiosity are more prevalent in individuals with manic symptoms, whereas persecutory and somatic delusions are equally prevalent across diagnostic categories (79). Other studies have shown that Schneiderian-type delusions (thought insertion, thought withdrawal, etc.) are not pathognomonic to schizophrenia and lack predictive diagnostic specificity when compared to other types of delusions (12, 80). By categorizing and examining the longitudinal course of delusions by either thought or thematic delusions, we showed that individuals with schizophrenia experience an increased prevalence and severity of thought delusions and to a lesser extent, increased thematic delusions when compared to individuals with affective psychoses. Thus, although thought and thematic delusions are more prevalent in individuals with schizophrenia, they are not pathognomonic. Although the presence of delusions themselves may persist within an episode of illness regardless of the diagnostic category, all types of delusions followed a remitting and relapsing course.

Additionally, we are the first study to report the probability of experiencing the same type of delusion in a subsequent evaluation over 20 years by diagnostic category. Interestingly, referential and thought dissemination delusions showed a higher probability of recurrence regardless of diagnosis; persecutory delusions showed a higher probability of recurrence in individuals with schizophrenia; and delusions of grandeur showed a higher probability of reoccurrence in individuals with affective psychoses. Importantly, we showed that individuals with affective psychoses, if experienced once, do not experience a recurrence of either thought withdrawal or “made” impulses. We also showed that in psychosis, referential, persecutory, and thought dissemination (mind-reading) delusions have a higher probability to recur and that “made” impulse, “made” volition, and self-deprecation delusions are the have the least probability to recur.

### Relationships between delusions, hallucinations, depression, anxiety, and negative symptoms

Our previous research have demonstrated that delusions have a higher probability to occur in conjunction with auditory verbal hallucinations (voices) and that voices together with thought delusions have a higher probability to be associated with increased disorganized thinking, cognitive, and depressive symptoms in comparison to voices with thematic delusional content (49). Building on this previous research, our current study investigated the relationships between thought vs. thematic delusions and hallucinations, depression, anxiety, and negative symptoms by diagnostic category over 20 years. Our data showed that the presence and severity of hallucinations (auditory, visual, tactile, and somatic) have the strongest association with the presence and severity of delusions when compared to depression, anxiety, or negative symptoms. Other studies have also shown that delusional content varies over time and are strongly associated with hallucinations and yet also occur independently or in association with other symptom clusters (81–83). Many researchers theorize that hallucinations and delusions exist along the same continuum and that the presence of hallucinations are directly related to the “meaning making” and the formation and maintenance of delusional reality (20, 28, 45, 49, 84). It is also pertinent to clinical practice and may even hold implications for mental health service provision: for example, those with the most severe hallucinations also tend to experience the most severe delusions, hence the potential need for extra clinical care and targeted interventions.

Negative symptoms have long been considered a hallmark symptom in individuals with schizophrenia (85, 86). However, the entanglement of positive symptoms (hallucinations and delusions) and negative symptoms is quite complex. Primary negative symptoms (e.g., avolition, anhedonia, asociality, blunted affect, and alogia) are a distinct intrinsic construct that is strongly associated with a poorer long-term outcome that occurs at a higher prevalence and persistence in individuals with schizophrenia (including deficit syndrome schizophrenia) and have been identified as a targeted research area in the psychosis prodrome and chronic trajectories (69, 87). Secondary negative symptoms, on the other hand, “are thought to be related to other factors, such as psychiatric or medical comorbidities, treatment adverse effects, or environmental factors” as a result of overwhelming internal (symptoms of psychosis, anxiety, depression) and external (social isolation as a coping mechanism and stigma) factors such as avolition and anhedonia arising from antipsychotic medications, or catatonic states as a severe consequence of post-schizophrenia depression (88). The entanglement of secondary negative symptoms associated with environmental factors requires additional research to delineate these factors. According to Bleuler, delusions are strongly associated with both negative symptoms and intense anxiety (89–93). In fact, the role of anxiety is deeply rooted in many symptoms of psychosis (94). Our longitudinal findings showed that negative symptoms are associated with the overall presence of delusions and that severe anxiety is associated with thought, thematic and overall delusions concur with previous cross-sectional findings.

The quantitative findings alone cannot capture the experience of delusional reality and the individuals' active engagement in making sense of the psychotic experience because quantitative measures typically are unable to facilitate a deeply nuanced explorative inquiry into the underlying individualized formation of a specific type of delusions. Quantitative findings such as ours can nonetheless support qualitative accounts and both types of methodology ought to work together and guide research into new and meaningful avenues. Although the “*experience of meanings*” is not investigated directly by qualitative methods in our current study, our results reflect the sheer complexities of, and entanglements between, various psychopathologies, all of which can directly impact an individual's meaning-making process and coherence in their sense of self. While gaps and distances remain between mechanisms, relationships, and meaning-making processes, we believe such an experience of meanings is an adequate snapshot of delusional realities. For example, Kusters (95) describes the lives of several individuals with psychosis who have interpreted, and navigated experiences otherwise classified as “delusions” by synthesizing the experience within the context of self, social, and cultural factors (95). Along these lines (96), conducted a systematic review of qualitative studies highlighting multiple themes, one of which examines the hyper-salient atmospheric changes in which the boundaries of self become porous, “At that moment, I believe you are in contact with the universe. Every step that I took was rhythmical, and after a while, everything I did was rhythmical, every step, every eye movement. You have a better feeling for timing in which you master each moment, a moment in which you lose time and space, hour and time (1, 96).” In a mixed-methods publication conducted by Jones et al. (48) examining agency and meaning-making in the formation and maintenance of delusional reality, the experience was described as “actually kind of interesting... the way that the delusions [and hallucinations] that I had were kind of teleological, in a certain sense. I feel like they weren't random. In a lot of ways, they were kind of fulfilling my own preconceived thoughts, desires—not always, but in a lot of ways, they had a purpose.” Individual interpretation and understanding of delusional realities are profoundly influenced within a cultural context and by prior history resulting in highly individualized meaning that is given to the experience “It is your whole life which is being revised (97, 98).”

## Conclusions

Delusional reality is a fundamental (if not ineffable) transformation in the experience of reality and the sense of being-in-the-world which is no longer embedded in the individual's familiar experiential milieu. In individuals with psychosis, delusional content can vary over time and typically manifests as a construct of multiple thought and/or thematic delusions that exist within a social structure. Given the significant associations between delusions and hallucinations, future research is needed to design better qualitative and quantitative measures that map onto the internal experience of delusional realities. These may be *via* participatory research which actively encourages the inclusion of individuals with psychosis in the design and delivery of research, through mixed methods to maximize the benefits of both quantitative and phenomenological/qualitative approaches. Additional research is also needed to investigate the influence of social factors and cultural contexts on the formation and maintenance of delusional reality. Our research emphasizes the need for future clinical interventions that specifically evaluate and target the co-existence and entanglement of delusions and hallucinations. Lastly, delusional reality is a “living” multimodal construct of sensory, perceptual, somatic, emotional, and social embodiment that manifests as an “*experience of meanings*.”

## Data availability statement

The raw data supporting the conclusions data cannot be shared due to concerns over consent/ethics. Further inquiries can be directed to the corresponding author/s.

## Ethics statement

The studies involving human participants were reviewed and approved by the University of Illinois at Chicago Institutional Review Board. The participants provided their written informed consent to participate in this study at index and at each subsequent follow-up.

## Author contributions

CR: conceptualization, investigation, methodology, data curation, formal analysis, writing—original draft, review and editing, and project administration. MH: funding acquisition, conceptualization, investigation, methodology, resources, writing—review and editing, and project administration. CH: conceptualization, writing—original draft, and review and editing. LT: methodology, data analysis, writing—review and editing. TJ: conceptualization, investigation, methodology, writing—original draft, and review and editing. HH: writing—review and editing. All authors contributed to and have approved the final manuscript.

## Funding

This work was supported in part by USPHS Grants MH-26341 and MH-068688 from the National Institute of Mental Health, USA (HH) and a Grant from the Foundation for Excellence in Mental Health Care G5014 (HH). The funding bodies had no other contribution to any part of the article.

## Conflict of interest

Author LT was employed by Advocate Aurora Health. The remaining authors declare that the research was conducted in the absence of any commercial or financial relationships that could be construed as a potential conflict of interest.

## Publisher's note

All claims expressed in this article are solely those of the authors and do not necessarily represent those of their affiliated organizations, or those of the publisher, the editors and the reviewers. Any product that may be evaluated in this article, or claim that may be made by its manufacturer, is not guaranteed or endorsed by the publisher.
