# Supplementary material for: ‘An experience of meaning’: A 20-year prospective analysis of delusional realities in schizophrenia and affective psychoses
Source: Front Psychiatry. 2022 Aug 4;13:940124. doi: 10.3389/fpsyt.2022.940124 (PMC9388349; doi:10.3389/fpsyt.2022.940124)
Supplement: Supplementary file 1 [file Table_1.DOCX]

**Supplemental Materials 1: Detailed description of Research Measures.**

***Individuals and measures***

***Diagnostic Indices***

Individuals were diagnosed at index hospitalization using the Diagnostic and Statistical Manual Version III criteria (American Psychiatric Association, 1980; Spitzer, Robert L., Endicott, & Robins, 1975). Additionally, the Schedule for Affective Disorders and Schizophrenia (SADS) (Endicott & Spitzer, 1978), the Schizophrenia State Inventory (Grinker Sr, Roy Richard Ed & Harrow, 1987) were administered as well as collateral information as part of the diagnostic determination (diagnostic criteria reliability was kappa 0.88).

***Measures of premorbid function***

Assessments were conducted by trained research assistants who were blind to diagnosis and previous ratings. At index hospitalization two major indices examining the prognostic potential of participants were examined using the Vaillant (Vaillant, 1962; Vaillant, 1978) and Stephen’s symptomatic Prognostic Index (Stephens, Richard, & McHugh, 1997; Westermeyer & Harrow, 1984) to predict the effects the prognostic potential versus moderate prognostic potential.  We also administered Zigler’s Prognostic Index (Zigler & Glick, 2001) based on developmental characteristics (e.g. graduated from high school).  These symptomatic and developmental indices allowed an estimation of the participants’ theoretical prognosis in that both prognostic schemes were collected at initial hospitalization and applied as prognostic variables to study later outcome results.

***Measures used to assess psychopathology***

Clinical symptoms of psychosis such as hallucinations and delusions were assessed using The Schedule for Affective Disorders and Schizophrenia (SADS interview: (Spitzer, R. L. & Endicott, 1978)). The presence and severity of psychopathology were rated on a 3 point scale with 1 indicating the absence of symptoms, 2 uncertainty about presence of symptoms, and 3 indicating the definitive presence of the symptom.

SADS questionnaire items for 16 Delusions measured at baseline and six follow-ups over 20 years.

1. Delusions of Reference: *Do people seem to drop hints about your or say things with a double meaning or do things in a special way so as to convey a meaning? Have things seemed especially arranged?*
2. Delusions of Thought Dissemination (belief that people can read his mind or know his thoughts): *Can people know what you are thinking (in some strange way)? (Not just by your facial expression?)*
3. Delusion of Control ~ “Made” feelings or emotions: *Do you have feelings or emotions which are not your own? That are put into you by some force or power you cannot control?*
4. Delusion of Control ~ “Made” impulses: *Do you have impulses that are not you own? Are you made to do things suddenly by some force or power that you can’t control?*
5. Delusion of Control ~ “Made” volitional acts: *Is someone else or something else making your movements or actions for you without your intention? Are you made to do or say things that you do not intend? What about your handwriting, is that controlled by someone or something outside of yourself?*
6. Delusions of Thought Broadcasting: *Did you ever feel that your thoughts were broadcast so that other people know what you were thinking?*
7. Delusions of Thought Insertion: *Did you feel that thoughts were put into your head that were not your own?*
8. Delusions of Thought Withdrawal: *Did you feel that thoughts were taken away from you by some external force?*
9. Persecutory Delusions: *Has anyone been making life hard, or deliberately causing you trouble, or trying to hurt you? (Is there some group involved?)*
10. Delusions of Self-deprecation (Guilt or Sin): *Have you felt that you committed a crime, or have done some terrible thing and deserved punishment?*
11. Grandiose Delusions: *Have you felt that you are particularly important person or that you have special powers or abilities?*
12. Somatic Delusions: *You mentioned that your (part of body) was giving you trouble. What do you think is really wrong? (Has your appearance changed?).*
13. Nihilistic Delusion: *Have you felt that something terrible will happen or has happened).*
14. Religious Delusions: *Religious delusions: Are you a very religious person? (Have you had any unusual religious experiences?)*
15. Sexual Delusions: *Rate if noted in general inquiry.*
16. Fantastic Delusions: *Rate if noted in general inquiry.*

***Measures of Hallucinations, Negative Symptoms, Anxiety and Depression***

Hallucinations, anxiety, and depression were obtained using the SADS and were evaluated at each timepoint. The presence and severity of multiple types hallucinations that included auditory, visual, olfactory, and somatic or tactile; items were rated on a 3-point scale ranging from the absence of any type of hallucination, suspected or likely presence of any type of hallucination, or definite presence of any type of hallucination. Anxiety as defined in the SADS is a measure of the subjective feelings of anxiety, fearfulness, or apprehension, excluding panic attacks, whether accompanied by somatic anxiety, and whether focused on specific concern of not. The presence and severity of anxiety were rated on a 6-point scale with 1 indicating the absence of symptoms and 6 indicating extreme, e.g., pervasive feelings of intense anxiety. Depression was based on feelings of depression (sad, blue, moody, down, empty, as if you didn’t care). The presence and severity of depression were rated on a 7-point scale with 1 indicating the absence of symptoms and 7 indicating very extreme, e.g., constant unrelenting extremely painful feeling of depression.

The presence and severity of Negative systems is based on a composite score of items from Strauss and Carpenter's Psychiatric Assessment Interview (PAI) (Carpenter, Sacks, Strauss, Bartko, & Rayner, 1976) and included the following domains 1.) Poverty of speech consisted of long lapses before replying to questions or responding to instructions, restriction of quantity of speech, individual fails to answer, questions must be repeated, and slowed speech. 2.) Flat affect consisted of avoids looking at examiner during interview, blank, expressionless face, reduced emotion shown when delusional or normal material is discussed which would usually bring out emotion, apathetic and uninterested, reduced changes in inflection of voice; monotonous voice or low voice, difficult to hear. 3.) Psychomotor retardation that consisted of slowed in movements, reduction in clear consciousness or voluntary movements (Pogue-Geile & Harrow, 1985). Each of the 13 scale items was rated as either: 0, absent; 1, present but in mild form; or 2, present in marked form. In addition to the individual subscales of poverty of speech, flat affect, and psychomotor retardation, a negative symptom total score was also computed as an estimate of the severity of the overall negative symptom syndrome, which was a sum of the subscale scores.

Supplemental References

American Psychiatric Association. (1980). *Quick reference to the diagnostic criteria from DSM-III* The Association.

Carpenter, W. T., Sacks, M. H., Strauss, J. S., Bartko, J. J., & Rayner, J. (1976). Evaluating signs and symptoms: Comparison of structured interview and clinical approaches. *The British Journal of Psychiatry, 128*(4), 397-403.

Grinker Sr, Roy Richard Ed, & Harrow, M. E. (1987). *Clinical research in schizophrenia: A multidimensional approach.* Charles C Thomas, Publisher.

Pogue-Geile, M. F., & Harrow, M. (1985). Negative symptoms in schizophrenia: Their longitudinal course and prognostic importance. *Schizophrenia Bulletin, 11*(3), 427-439.

Spitzer, R. L., Endicott, J., & Robins, E. (1975). Clinical criteria for psychiatric diagnosis and DSM-III. *The American Journal of Psychiatry.*

Spitzer, R. L., & Endicott, J. (1978). Schedule for affective disorders and schizophrenia (SADS), New York, New York state psychiatric institute. *Biometrics Research.*

Stephens, J. H., Richard, P., & McHugh, P. R. (1997). Long-term follow-up of patients hospitalized for schizophrenia, 1913 to 1940. *The Journal of Nervous and Mental Disease, 185*(12), 715-721.

Vaillant, G. E. (1962). The prediction of recovery in schizophrenia. *The Journal of Nervous and Mental Disease, 135*(6), 534-543.

Vaillant, G. E. (1978). A 10-year followup of remitting schizophrenics. *Schizophrenia Bulletin, 4*(1), 78.

Westermeyer, J. F., & Harrow, M. (1984). Prognosis and outcome using broad (DSM-II) and narrow (DSM-III) concepts of schizophrenia. *Schizophrenia Bulletin, 10*(4), 624-637.

Zigler, E. F., & Glick, M. (2001). *A developmental approach to adult psychopathology* John Wiley & Sons.
