# Supplementary material for: ‘An experience of meaning’: A 20-year prospective analysis of delusional realities in schizophrenia and affective psychoses
Source: Front Psychiatry. 2022 Aug 4;13:940124. doi: 10.3389/fpsyt.2022.940124 (PMC9388349; doi:10.3389/fpsyt.2022.940124)
Supplement: Supplementary file 2 [file Table_2.DOCX]

**Supplemental Material 2**

***Demographic Characteristics***

As shown in Table 2, the sample consisted of a total of 262 individuals with one or more follow-up who met diagnostic criteria for schizophrenia (*n=151*) or affective psychosis (*n=111; psychotic depression n=52 and bipolar psychotic n=59*). The mean age at index hospitalization for the sample was 23. There were no significant diagnostic differences found in race, age at first episode, age at index hospitalization, social class, number of previous hospitalizations, or number of follow-up evaluations. However, there was a significant diagnostic difference in sex (*X*(1)=8.46, p=0.004) showing a higher percentage of male participants with schizophrenia. There was also a significant diagnostic difference found in the level of education (*t*(251)=2.69, p=0.008) showing that individuals with schizophrenia had a lower level of education. Additionally, there was a significant difference in prognostic indices showing that individuals with schizophrenia had worse prognostic indices as measured by Valliant-Stevens scores at index hospitalization (*t*(212) = 5.67, *p* ≤ 0.001). When all groups are combined, 88% of the participants had experienced their first episode of symptoms at ≤ 23yrs. Overall, 66% of the sample had 4 or more follow-up evaluations.
